# HPV testing in Polish population-based cervical cancer screening programme (HIPPO project)—study protocol of a randomised healthcare policy trial

**DOI:** 10.1186/s12885-023-11597-5

**Published:** 2023-11-17

**Authors:** Patrycja Glinska, Katarzyna Komerska, Beata Janik, Julia Olkowicz, Ilona Jedrzejewska, Anna Macios, Paulina Wieszczy, Michal F. Kaminski, Marc Arbyn, Andrzej Nowakowski

**Affiliations:** 1 Department of Oncological Gastroenterology, Maria Sklodowska-Curie National Research Institute of Oncology, Roentgen Street 5, Warsaw, Poland; 2 Warsaw PhD School in Natural and BioMedical Sciences, Maria Sklodowska-Curie National Research Institute of Oncology, Roentgen Street 5, Warsaw, Poland; 3 Department of Cancer Prevention, Maria Sklodowska-Curie National Research Institute of Oncology, Roentgen Street 5, Warsaw, Poland; 4 National Institute of Geriatrics, Rheumatology and Rehabilitation, Spartanska Street 1, Warsaw, Poland; 5 Doctoral School of Translational Medicine, Centre of Postagraduate Medical Education, Marymoncka Street 99/103, Warsaw, Poland; 6 Department of Gastroenterology, Hepatology and Clinical Oncology, Centre of Postgraduate Medical Education, Roentgen Street 5, Warsaw, Poland; 7 Clinical Effectiveness Research Group, Institute of Health and Society, University of Oslo, Forskningsveien Street 3A, Oslo, Norway; 8 Department of Health Management and Health Economics, University of Oslo, Forskningsveien Street 3A, Oslo, Norway; 9 Unit of Cancer Epidemiology, Belgian Cancer Centre, Sciensano, J. Wytsmanstreet 14, B1050 Brussels, Belgium

**Keywords:** Screening programme, hrHPV test, Cytology, Cervical cancer, Poland

## Abstract

**Background:**

An Organised Cervical Cancer Screening Programme (OCCSP) was started in Poland in 2006/2007. Each woman aged 25 to 59 is eligible for a free Pap test every 3 years in OCCSP. Despite implementation of the OCCSP, the age-standardised cervical cancer (CC) incidence and mortality rates in 2019 were 7.3/100 000 and 3.9/100 000 respectively and were still higher than those in Western European countries with well-organised screening programmes. Apart from low coverage of the OCCSP, suboptimal performance of the screening test (conventional cytology) may be partially responsible for this situation. Several countries have already incorporated high risk Human Papillomavirus (hrHPV) testing in CC screening as a more sensitive tool reducing the risk of missing precancerous lesions and allowing for extension of screening intervals. The European Guidelines for Quality Assurance in Cervical Cancer Screening recommend pilot evaluation of a new screening test in country-specific conditions before its implementation.

**Methods:**

The HIPPO project (HPV testing In Polish POpulation-based cervical cancer screening program) is a randomised health services study nested in the OCCSP in Poland. The project will randomise 33 000 women aged 30–59 years to cytology or hrHPV testing (ratio: 1:1) with age stratification. In the cytology arm women with repeated Atypical Squamous Cells of Undetermined Significance (ASC-US) or ≥ Low–Grade Squamous Intraepithelial Lesions (LSIL) are referred for colposcopy. In the other arm, hrHPV ( +) women with ≥ ASC-US reflex Liquid-Based Cytology (LBC) are referred for colposcopy. Primary endpoints include detection rates of histologically confirmed high grade intraepithelial lesions or worse (CIN2 +) in each arm.

**Discussion:**

This pilot randomised healthcare study nested in the OCCSP in Poland will assess and compare the performance of hrHPV testing to current standard—cytology in order to make decisions on implementation of HPV-based screening in the country.

**Trial registration:**

This randomised healthcare service study was prospectively registered at https://clinicaltrials.gov/ (identifier: NCT04111835, protocol ID 28/2019) on 19th of September 2019.

## Background

In 2006/2007 an Organised Cervical Cancer Screening Programme (OCCSP) was implemented in Poland. It comprised personal invitations of women aged 25–59 years to have a screening Pap test every three years. By the decision of the Ministry of Health related to high costs, uncertain effectiveness and issues with access to personal data, the postage of personal invitations for cytology was stopped in 2016. Currently, Pap test collection in the Programme is available in 1790 gynaecological clinics across the country. Additionally, in 2011 family medicine practises employing certified midwives entered the Programme and 103 of them are currently actively collecting Pap smears. A modified Bethesda system (TBS) for evaluation and reporting of cytological results and a protocol derived from European Guidelines and Recommendations of the Polish Gynaecological Society is currently in practice [1–3]. In 2021, cytology was assessed in 74 laboratories around the country working within the OCCSP. Colposcopy in the Programme was performed in 62 clinics. The costs of medical procedures are reimbursed completely by the National Health Fund (NHF). The costs of quality assurance and coordination of the Programme are covered by the Ministry of Health. Data collected in the OCCSP are stored in the Electronic Screening Database called SIMP (pl.: System Informatyczny Monitorowania Profilaktyki). Quality assessments of the Programme are undertaken by the Central Coordination Centre based at the Maria Sklodowska-Curie National Research Institute of Oncology in Warsaw every year.

Recent data indicate a possible acceleration of the decreasing trend in the burden of cervical cancer (CC) after implementation of the OCCSP [4]. However, despite implementation of the Programme, there were still 2407 new cases (World Age Standardised Rate 7.3/100 000) and 1569 deaths due to CC (World Age Standardised Rate 3.9/100 000) in 2019 in Poland [5]. These rates are still higher than in Western European countries with well-established cancer prevention programmes. The factors explaining this unfavourable situation are low participation rate in the OCCSP – coverage of 14% reported in 2019, possible suboptimal performance of the Programme procedures including the screening test (conventional cytology) and triage and the very popular concurrent opportunistic screening of unknown coverage and quality [6]. Conventional cytology is the main primary screening test in the OCCSP. Liquid-based cytology (LBC) is available in a very small proportion.

Several countries have switched to screening with high risk Human Papillomavirus (hrHPV) testing because of its higher efficacy [7, 8]. The main advantage of HPV-based testing over cytological screening is its high sensitivity which reduces the risk for missing precancerous lesions and allows for extension of intervals between screening tests to five or more years [8]. According to the European Guidelines [2, 9], HPV-based screening programmes should be thoroughly evaluated in pilot studies before implementation. Since specificity of HPV testing is lower than that of cytology, more positive results are expected, with higher detection rates of high-grade lesions, but also of low-grade lesions requiring appropriate triage to avoid an increased burden of follow-up. European Guidelines recommend HPV testing alone and do not advocate HPV co-testing with cytology, because of the high costs, higher referral rate for an extremely small gain in protection [2, 8]. Triage of HPV positive women is still a subject of research and various algorithms are proposed [7]. HPV-based screening has been fully implemented in the Netherlands and women with positive test results are tested by reflex LBC twice in a 6-month interval in case of a first negative LBC sample [10]. A positive LBC result triggers colposcopic assessment.

Recently, new data appeared, e.g. on the use of immunohistochemical or molecular methods in triage of HPV positive women [7, 11]. The CINtec® PLUS Cytology test is a triage method for CC screening and simultaneously detects overexpression of p16 and Ki-67 which indicates transforming HPV infections [12]. The QIASURE methylation test is aimed at detecting hypermethylation of the promoters of the FAM19A4 and hsa-mir 124–2 genes [13]. Methylation of these genes indicates an ongoing carcinogenic process. Lack of methylation, in turn, indicates a low short-term risk of developing cancer. To our knowledge, performance of the two triage tests have not yet been compared prospectively in a screening programme.

## Aim

The HIPPO project (HPV testing In Polish POpulation-based cervical cancer screening program) was set up to evaluate and compare performance of an investigational screening strategy: i) HPV molecular testing with ii) current practice – conventional cytology/LBC in everyday clinical practice of the OCCSP in Poland. We also aim to compare performance of the CINtec® PLUS Cytology test and QIASURE methylation test as triage methods for HPV positive, twice LBC negative women in a screening programme.

## Methods

**Fig. 1:**
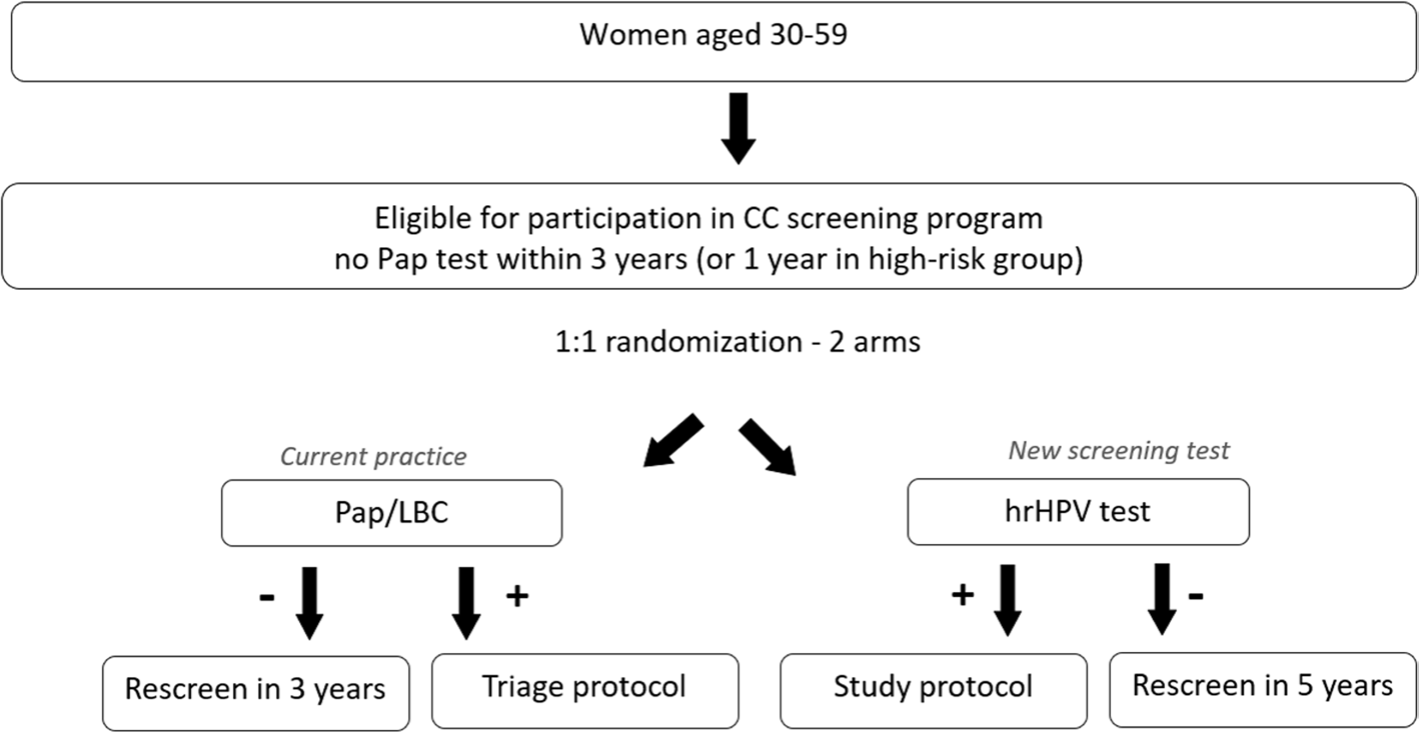
The flowchart of women enrolled in the HIPPO trial. ASC-US—Atypical Squamous Cells of Undetermined Significance. CC – cervical cancer. hrHPV test—high risk Human Papillomavirus test. LBC—Liquid-Based Cytology. LSIL—Low-Grade Squamous Intraepithelial Lesion. NILM—No Intraepithelial Lesion or Malignancy

The project is designed as a randomised healthcare intervention nested in everyday practice of the OCCSP in Poland. It involves randomization of women attending screening into two arms according to the primary screening test applied: cytology (conventional or LBC, depending on the local site) and hrHPV test in a 1:1 ratio and age stratification into three strata: 30–39, 40–49 and 50–59 years. Since HPV testing in screening is not recommended for women younger than 30 years of age the project includes women aged 30–59 years [2]. The scheme of the project is presented in Fig. 1.

Inclusion criteria to the project are: i) age 30–59 and ii) without screening Pap test within the preceding three years (or one year for women with risk factors: HR HPV infection/HIV infection/use of immunosuppressive drugs entitled to annual screening based on information from the SIMP) in the OCCSP. Exclusion criteria for study are: the Pap test taken within the preceding three years in the OCCSP and illegibility for screening according to SIMP (previous treatment, hysterectomy etc.).

Women attending the selected screening sites are informed on the project by trained site personnel using pilot-tested information brochures with detailed information about the study, objectives of the study, duration, procedures, potential risks and benefits. By the decision of the Ministry of Health in Poland signing an informed consent is required from women to participate in the study. Women who accept participation are randomised by the SIMP to the competing screening tests and strategies. The sample takers and researchers involved in data collection and monitoring are aware of the arm to which each participant is assigned. The patients are informed about which arm they are also assigned to and they cannot change their arm. A questionnaire with screening history (participation in opportunistic screening, treatment of cervical lesions, HPV vaccination status etc.) are collected from participating women.

The primary endpoint is the detection rate (DR) of histologically confirmed CIN2 or higher in each screening arm and detection rate ratio (DRR) of CIN2 + in HPV test *vs* cytology arm in intention-to-treat (ITT) analysis. Secondary endpoints include detection rate ratio of CIN1 + , CIN3 + (including adenocarcinoma in situ) and invasive CC in both ITT and per-protocol (PP) analysis, restricted to women who adhered to the foreseen follow-up.

Other outcome measures are: (1) distribution of women by the screening test results; (2) referral rate for colposcopy; (3) compliance with referral for colposcopy; (4) positive predictive value of referral for colposcopy calculated for each screening test and separately by histology results (no CIN, CIN1, CIN2, CIN3, AIS, CIN2 + , CIN3 + , invasive cancer (squamous/adenocarcinoma)); (5) DR of histologically confirmed CIN2 + in patients with hrHPV positive test, two NILM LBCs six months apart and a positive CINtePlus Cytology and/or Qiasure test; (6) DRR of histologically confirmed CIN2 + after CINtec® PLUS Cytology test vs. QIASURE methylation test; (7) yield of use of CINtec® PLUS Cytology test vs. QIASURE methylation test in terms of detection rate of histologically confirmed CIN2 + .

Patients are recruited by the Cervical Cancer Prevention Clinic at the Department of Cancer Prevention at the Maria Sklodowska-Curie National Research Institute of Oncology in Warsaw (main centre) and 8 more sites from distant regions of Poland (Lublin, Masovian, Silesian, Swietokrzyskie and West Pomeranian Voivodeship). There are 24 gynaecological clinics, 9 diagnostic (cytological&molecular) laboratories and 10 colposcopy clinics. All sites are selected through a tendering process and have to fulfil several criteria for inclusion into the project (active participation in the OCCSP, being certified for HPV testing with the chosen clinically validated hrHPV DNA assays)to obtain results representative for the entire country.

The hrHPV tests are performed in diagnostic centres: Maria Sklodowska-Curie National Research Institute of Oncology (Roentgen Street 5, Warsaw, Armii Krajowej Street 15, Gliwice (branch in Gliwice)), Holy Cross Cancer Centre (Prezydent Stefan Artwinski Street 3, Kielce), Medical Center – Diagnostic (Niklowa Street 9, Siedlce), ALAB Laboratories (Stepinska Street 22/30, Warsaw), Non-public Health Care Center MEDITEST Medical Diagnostics (Bronislawy Street 14D, Szczecin), Non-public Health Care MULTIMED Gynecology and Obstetrics (Monte Casino Street 13, Koszalin), Non-Public Health Care Department of Pathomorphology ALFAMED. Edward Cwierz, Maciej Cwierz (Jana Kilinskiego Street 78, Zamosc), Femina. Medical Centre (Klodnicka Street 23, Katowice).

Site coordinators are contracted to monitor patients with abnormal screening test results in both arms. They are responsible for full acquisition and registration of histological outcomes. Coordinators regularly provide detailed reports to the main centre—Cancer Prevention Centre.

Additionally, biobanking of cytological samples taken from patients participating in the HIPPO project is performed in the Maria Sklodowska-Curie National Research Institute of Oncology for the purpose of potential future retrospective analyses if new CC screening or triage technologies emerge.

The study groups will be compared using two-sided tests: chi square test or analysis of variance (ANOVA, Kruskall-Wallis test). A chi-square test will be used to compare the characteristics of patients in different groups. The Kruskal–Wallis test will be used to compare differences in variables between two groups. If variations exist, a logistic regression analysis will be performed to permit control of potential confounding factors. The statistical significance in the experimental group compared with control will be analysed by ANOVA. Results will be considered statistically significant at the 0.05 significance level. The groups will be characterised by descriptive statistics: cardinality and percentage for discrete variables, mean and standard deviation for continuous variables with normal distribution, median and interquartile range for continuous variables with different distribution. The distribution of variables will be assessed among the groups. If variations exist, a logistic regression analysis will be performed to permit control of potential confounding factors. All analyses will be carried out with Stata 14.1 [14].

The study hypothesis assumes that the detection rate of histologically confirmed CIN2 + is significantly higher when HPV HR tests are used for screening in comparison to current standard of cytology (conventional or LBC).

There is scarce data on histological endpoints of the Polish OCCSP since the majority of colposcopy and biopsy procedures are performed outside the program and their results are not registered in the screening database. However, data on cytological results is fully available and credible. The data regarding the rate of high‐grade abnormalities (≥ ASC-H) results were extracted from the polish screening database. Some of them were conducted at OCCSP and had histopathological confirmation. Data extraction was used in analysis. Based on it, the rate of ASC-H or worse results is about 0.5% among screening Pap smears; therefore 0.5% is assumed as the baseline DR of CIN2 + in the control arm. Updated meta-analyses showed a relative sensitivity for CIN2 + of HPV test vs cytology of around 1.5 [8, 15–17]. As a consequence, the hypothesised DR of CIN2 + in the experimental HPV screening arm is 0.75%. Accepting the above assumptions, an alpha error of 5% and a power of 80%, each of the study arms should include around 16 500 women.

Based on SIMP, we calculated that the predicted referral rate for colposcopy is 1.3% and CC prevalence is 0.0134% in cytology arm.

### Control arm – current practice

**Fig. 2:**
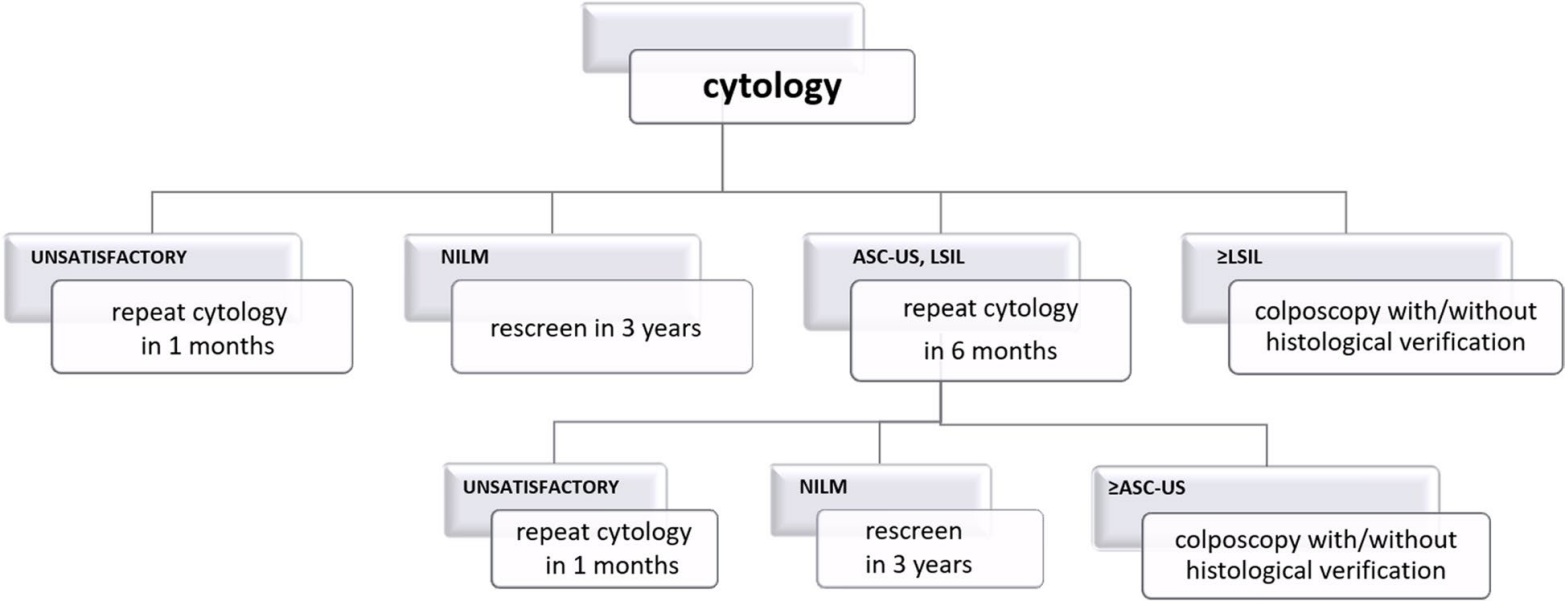
Algorithm of control arm. ASC-US—Atypical Squamous Cells of Undetermined Significance. CC – cervical cancer. hrHPV test—high risk Human Papillomavirus test. LBC—Liquid-Based Cytology. LSIL—Low-Grade Squamous Intraepithelial Lesion. NILM—No Intraepithelial Lesion or Malignancy

Conventional cytology (or LBC in our site) is the screening tool used in this arm. Triage algorithm is based on Recommendations of the Polish Gynaecological Society [18]. The smears are interpreted according to a modification of the TBS. Women with abnormal Pap smear results are supposed to be triaged within the Programme with 1) repeated Pap smear for atypical squamous cells of undetermined significance (ASC-US), low grade squamous intraepithelial lesion (LSIL) or 2) colposcopy (with or without targeted biopsy) for repeated ASC-US and LSIL, or first observation of atypical squamous cells – cannot exclude high grade squamous intraepithelial lesion (ASC-H), high grade squamous intraepithelial lesion (HSIL), atypical glandular cells (AGC), squamous cell carcinoma (SCC), adenocarcinoma in situ (AIC). If the screening cytology result is negative, rescreening is scheduled after 3 years. Medical records of procedures performed in the OCCSP are stored in the central electronic database (SIMP). hrHPV testing recommended for triage of ASC-US and LSIL [8] is not available in the Programme but can be performed within NHF-reimbursed gynaecological services. The scheme of the algorithm is presented in Fig. 2.

Cervical cytology samples taken by the midwives or gynaecologists are collected using a cervical brush and placed on a glass slide for conventional cytology or in SurePath medium (BD Totalys Slide Prep, Becton Dickinson TriPath) for LBC [19].

Currently, facilities participating in the OCCSP are mandated to monitor patients' outcomes and actively invite women with abnormal results from the initial stage examination for further in-depth diagnostics. However, due to organisational challenges and a lack of effective oversight, monitoring is not working very well.

### New screening test and triage algorithm – hrHPV test arm

Clinically certified hrHPV tests in the experimental arm must fulfil Meijers criteria [20] and must have been validated in Valgent platform [21]. All used assays allow the detection of two HPV genotypes (16, 18) and the identification of twelve additional genotypes (31, 33, 35, 39, 45, 51, 52, 56, 58, 59, 66 and 68). hrHPV testing is performed with the Cobas 4800 HPV Test (Roche Molecular System, Pleasanton, CF, USA), INNO-LiPA HPV Genotyping Extra II assay (INNO-LiPA; Fujirebio Europe, Ghent, Belgium), Onclarity HPV Assay (BD Diagnostics, Sparks, MD, USA), Harmonia HPV (Liferiver, Shanghai, China), AmpFire Multiplex HPV Assay (Atila BioSystems, Mountain View, CA) on the BD SurePath which complies with the International Guidelines for HPV Test Requirements for Cervical Screening. Results are reported as HPV negative, HPV positive (HPV16, HPV18 and/ or other HPV types) or invalid. Cervical samples are taken by a gynaecologist or a certified midwife in OCCSP in Poland.

Women with HPV-negative results are released for the next round with a five-year interval. If hrHPV is detected, a reflex LBC on the same sample is conducted. HPV-positive women with abnormal cytology (ASC-US or worse) are directly referred for colposcopy with mandatory biopsy (punch biopsy, or Large loop excision of the transformation zone (LLETZ), with or without endocervical curettage upon decision by the colposcopist). The referral is automatically issued by the SIMP for ≥ ASC-US. Patients are informed by medical staff about the colposcopy reference centres in the OCCSP. If histological results by any means are obtained outside the OCCSP the site coordinators are responsible for full acquisition and registration of histological outcomes.

**Fig. 3:**
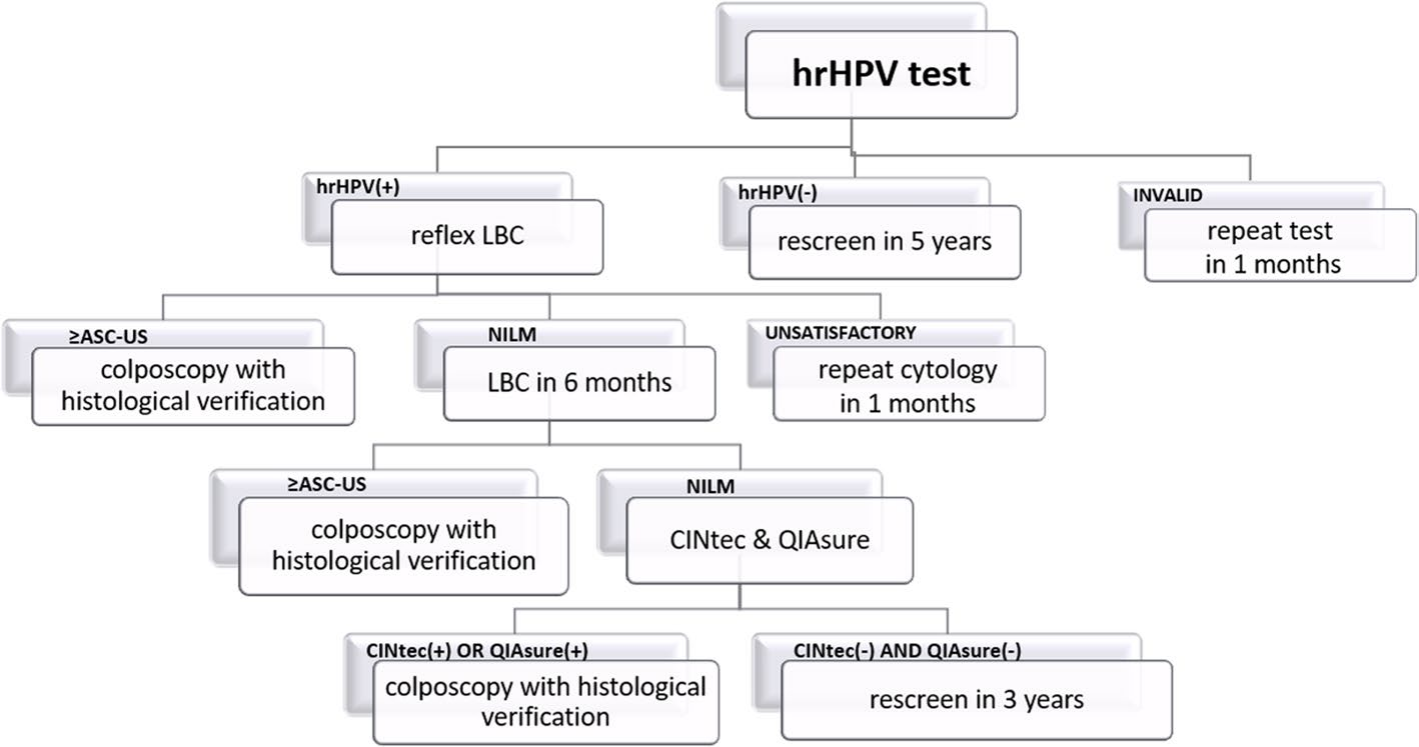
Algorithm of hrHPV arm. ASC-US—Atypical Squamous Cells of Undetermined Significance. CC – cervical cancer. hrHPV test—high risk Human Papillomavirus test. LBC—Liquid-Based Cytology. LSIL—Low-Grade Squamous Intraepithelial Lesion. NILM—No Intraepithelial Lesion or Malignancy

hrHPV-positive, reflex LBC-negative women are invited to LBC retesting after 6 months. After six months women with ASC-US or higher abnormality are referred for colposcopy with necessary biopsy. In hrHPV-positive and two subsequent NILM results, CINtec® PLUS Cytology tests and the QIASURE methylation tests are performed. If the result of at least one of them is positive, the patient is referred for colposcopy with biopsy. If both tests are negative, the woman returns in a shortened screening interval in 3 years. The scheme of the hrHPV arm is presented in Fig. 3.

Patients had planned visits according to the study algorithm:

- Visit 1: screening test (*test hrHPV* ± *LBC).*
- Visit 2': colposcopy with biopsy (*women with hrHPV (* +*),* ≥ *ASC-US in screening test*),
  - Visit 2’’(after 6 month): LBC *(women with hrHPV (* +*) and NILM in screening tes*t).
- Visit 3’: colposcopy with biopsy (*women with hrHPV (* +*), NILM and* ≥ *ASC-US in LBC after 6 months*).
  - Visit 3’’: colposcopy with biopsy (*women with HPV (* +*), double and CINtec PLUS Cytology (* +*) and/or QIASURE Metylathion test (* +*)*).

All reports of visits and results are available in the SIMP.

The purified DNA material after HPV test is banked and stored at − 80 °C. CINtec® PLUS Cytology (p16/Ki67 cytology dual staining) and QIASURE methylation test are performed on the residual SurePath cell-pellet.

## Discussion

Population-based cytological screening has the potential to reduce CC incidence and mortality through identification and treatment of precancerous lesions. However cytology is not a perfect screening test due to its limited sensitivity, and subsequent risk of interval cancers, in particular of adenocarcinoma.. A pilot audit of false negative cytological results was conducted in Poland and revealed a considerable burden of false negative Pap smears which resulted in interval cancers [6].

Identification of hrHPV as the aetiological factor of CC enabled elaboration of very sensitive molecular technologies which may replace cytology as a primary screening test and potentially provide better outcomes of screening. A number of randomised controlled trials comparing hrHPV testing to cytology, specifically the detection of high grade CIN (CIN 2 + and CIN 3 +), have been executed [22–25]. Based on meta-analyses, hrHPV primary screening has higher sensitivity than cytology primary screening in detecting precancerous cervical lesions (CIN2 + and CIN3 +), so it gives increased protection against development of CC [2, 26]. Due to the higher cross-sectional and longitudinal sensitivity, longer intervals and decreasing costs of hrHPV testing on a large scale, molecular screening has been proven to more effective and cost-effective than cytology and should be considered for implementation in screening programmes [8, 27]. The disadvantage of hrHPV testing is that it is less specific than cytology and therefore requires appropriate triage [28]. So according to the European guidelines for quality assurance in CC screening, before implementation of hrHPV primary testing to routine population-based CC screening programme, it is recommended to conduct pilot evaluation to ensure that an appropriate balance between harms and benefits may be achieved [2]. Recently the Netherlands, Turkey, England and Australia have implemented primary hrHPV-based screening programmes. Sweden, Finland, Germany and Italy have regional hrHPV primary testing or are at the roll-out phase. Some other countries are in the process of transition from cytology-based to hrHPV-based screening [29]. Management of hrHPV positive women remains a challenge for clinicians since no optimal, “gold-standard triage protocol” has been developed. Many triage strategies are being tested, however amongst them, two most common ones can be distinguished. First is based on performing reflex LBC after detection of any hrHPV type (Netherlands) and second uses HPV 16/18 genotyping to identify women with the highest risk for high-grade precancerous lesions and the need for direct referral for colposcopy (Australia, Italy) [22, 30].

The Netherlands was the first country to switch to hrHPV screening (January 2017). The algorithm of the HIPPO project is largely based on the Dutch CC screening programme since at the time of the development of our protocol, the Dutch programme was the only standardised hrHPV-based algorithm and used routinely in everyday clinical screening practice. In order to obtain final histological endpoints and to increase the sensitivity of mere colposcopy (without biopsy) commonly performed in Poland, we have implemented mandatory collection of at least one tissue sample (upon decision of the colposcopist) during colposcopy in the hrHPV arm. We have also decided to shorten the interval to 3 years for hrHPV-positive, twice LBC-negative women since in those women we consider the risk of CIN3 + development higher than in hrHPV-negative individuals.

In 2018 (after the first 18 months), the Dutch programme analysed referral for colposcopy and detection rate of CIN in women in screening based on hrHPV and cytology (tests performed between January 2015 to March 2016, before implementation of new screening programme). The result was, as expected, higher CIN2 + detection rate in the new hrHPV-based screening than in the cytology-based programme before [31]. In addition, more low-grade lesions were found in the newer compared to the older programme. Therefore, molecular screening may result in an increased number of unnecessary colposcopies, treatments, and adverse effects, particularly in younger women [31]. Among women aged ≥ 35 years, HPV primary screening entails only a limited increase in overdiagnosis of non-progressive lesions compared with cytology primary screening. The ATHENA trial supports the triage based on genotyping and direct referral of HPV16/18 positive women for colposcopy since they have the highest risk of CIN3 + diagnosis [23]. Australian [32] and Italian [29] Cervical Screening Programme algorithms are also based on HPV testing and genotyping for initial triage. However these programmes were initiated after the Dutch HPV-based screening programme on which we based the HIPPO protocol. American Society for Colposcopy and Cervical Pathology (ASCCP) decided to assist healthcare professionals with management of women attending CC screening and developed an evidence-based management guideline-mobile application stratifying risk of CIN3 + based on several pieces of data. However it was also launched when the work on the HIPPO protocol was well advanced and it was not included in our triage strategy.

Recently new data emerged [33] and FDA approval [34] was granted to novel biomarkers for use in triage of hrHPV-positive women. The CINtec® PLUS Cytology test is the first Food and Drug Administration (FDA) approved test to triage women with positive results of hrHPV test. The QIASURE methylation test (QIAGEN) has gained attention from researchers and data on its potential usefulness in triage of hrHPV women [33, 35]. Therefore in 2020 we amended the protocol of the HIPPO project and decided to compare the performance of these two tests in identification of high-grade cervical lesions in hrHPV positive and twice LBC negative women. Women who are tested positive on either of these tests are referred to colposcopy with mandatory histological verification. Women who test negative on both tests will be rescreened with hrHPV test in three years. The project will enable verification of the diagnostic yield of these two tests in detection of histologically confirmed CIN2 + in women with hrHPV-positive results and two 6-months apart NILM Pap results prospectively which has not been yet investigated. Data gathered in the HIPPO project will be subject to extensive analysis. Results will be important for clinical decision-making and setting-up appropriate algorithms. Data will be submitted to the Polish Health Technology and Tariff Agency for cost-effectiveness evaluation to guide decision-makers on the potential switch to molecular-based testing. Altogether this should facilitate the identification of a robust CC screening strategy in a reimbursed programme based on local evidence. Hopefully the introduction of HPV-based screening will accelerate the decreasing trends in CC burden in the country.

## Strengths and limitations of the study

This is the first randomised controlled trial to evaluate and compare performance of an investigational screening strategy: HPV molecular testing with cytology (conventional cytology/LBC) in everyday clinical practice in the OCCSP in Poland.

This trial will clarify whether screening using HPV testing detects more CIN2 + than routine screening based on cytology.

A major strength of this study is the large group of included patients in both screening arms.

The limitation may be selected population. Women can have current screening test results in private care.

Expectation bias may observe in the study. Medical staff has information which patients are participated in the project (and cytology/hrHPV arm).

The assumption that all ASC-H or worse will lead to a diagnosis of CIN2 + upon final histological examination is not accurate. This rate can be lower, but LSIL and ASC-US results may also manifest as CIN 2 + [36–38].

## Timelines

**October 2019** – start of the project at the Maria Sklodowska-Curie National Research Institute of Oncology, Warsaw, Poland.

**July 2020** – implementation of the Amendment to the protocol.

**November 2020** – introduction of CINtec® PLUS Cytology test and QIASURE methylation test.

**April 2021** – selection of additional five sites for execution of the project and signing contracts with the Maria Sklodowska-Curie National Research Institute of Oncology, Poland.

**September 2021** –inclusion of additional three sites from several distant regions of Poland into the project.

**November 2022** – planned completion of the recruitment for screening tests.

**December 2022** – December 2023 – planned completion of triage and final diagnosis of women with positive screening test results.

**2023/2024** – evaluation and publication of the final results of the project.

The interim analysis was performed on medical records of women involved in the HIPPO study between its start on October 28th, 2019, and September 27th, 2020. For the women included in the analysis the follow-up was stopped on November 30th, 2020. At the time the project was conducted in only one site (Maria Sklodowska-Curie National Research Institute of Oncology). Detailed data regarding abnormal results in each arm was retrieved from the SIMP database. Both intention-to-treat and PP analyses were performed accordingly to fixed endpoints. The results of the first interim analysis were presented during the EUROGIN 2021 virtual conference on May 31^st^, 2021.

## Extension phase

Extension phase to the time of the second screening round – 3 years after conventional cytology and LBC and 5 years after HPV testing is possible unless a prior decision on the roll-out of the HPV based screening nationwide would be made.

Assessment of similar and additional end-points after the second round of screening would be most valuable.

## Data Availability

Screening test and colposcopy (with histopathology) results and are available in the SIMP database. The SIMP is a central electronic database. Currently, it collects information and enables analysis of results of screening tests. The SIMP is linked with the treatment databases of the National Health Fund (NHF) and allows selection of patients who are eligible for preventive examinations. The data that support the findings of this study are available on request from the corresponding author, PG. The data are not publicly available due to containing personal information that could compromise the privacy of research participants.

## Abbreviations

ADC: Adenocarcinoma in situ
AGC: Atypical glandular cells
ASCCP: American Cancer Society, American Society for Colposcopy and Cervical Pathology
ASC-H: Atypical squamous Cells-Cannot Exclude High-Grade Squamous Intraepithelial Lesion
ASC-US: Atypical Squamous Cells of Undetermined Significance
CIN: Cervical intraepithelial neoplasia
CIN1 +: Cervical intraepithelial neoplasia grade 1 or worse
CIN2 +: Cervical intraepithelial neoplasia grade 2 or worse
CIN3 +: Cervical intraepithelial neoplasia grade 3 or worse
CC: Cervical cancer
DR: Detection rate
DRR: Detection rate ratio
hrHPV: High risk Human Papillomavirus
HSIL: High grade squamous intraepithelial lesion
ITT: Intention-to-treat
LBC: Liquid-Based Cytology
LLETZ: Large loop excision of the transformation zone
LSIL: Low–Grade Squamous Intraepithelial Lesion
NHF: National Health Fund
NILM: No Intraepithelial Lesion or Malignancy
OCCSP: Organised Cervical Cancer Screening Programmes
PP: Per-protocol
PPV: Positive predictive value
RCT: Randomised controlled trials
SCC: Squamous cell carcinoma
SIMP: (pol.: System Informatyczny Monitorowania Profilaktyki), the Electronic Screening Database
TBS: A modified Bethesda system
